# Socio-economic vulnerability and deaths of despair in Brazilian counties

**DOI:** 10.1016/j.pmedr.2024.102623

**Published:** 2024-01-20

**Authors:** Wellington Roberto Gomes de Carvalho, Karina Cardoso Meira, Aline Grimberg Pereira de Medeiros, Luise Bernardes da Silva Neves, Nicole Almeida Vardiero, Raphael Mendonça Guimarães

**Affiliations:** a Federal University of Triângulo Mineiro, Institute of Health Sciences, Department of Collective Health, Uberaba, MG, Brazil; b Federal University of Rio Grande do Norte. Health School, Natal, RN, Brazil; c Oswaldo Cruz Foundation, Center for the Integration of Data and Knowledge for Health. Salvador, BA, Brazil; d IDOMED/Estácio de Sá University, School of Medicine. Rio de Janeiro, RJ, Brazil; e Oswaldo Cruz Foundation, National School of Public Health, Department of Social Sciences. Rio de Janeiro, RJ, Brazil

**Keywords:** Socioeconomic deprivation, Deaths of despair, Social Determinants of Health, Economic recession, Mortality, Suicide

## Abstract

Over the past few years, there has been a progressive increase in premature deaths attributable to suicide, drug overdose, and alcohol-related liver disease that impact life expectancy. Regarding the relationship with contextual effects, the evidence is developing, especially in countries with a peripheral economy, as is the case of Brazil. We carried out an analysis aimed at estimating the relationship between socioeconomic insecurity and deaths due to despair in Brazilian cities. We used 5,570 counties' data to create clusters concerning socioeconomic development and then analyzed age-adjusted mortality rates (ASMR) from each of them and compared them using the ANOVA test. Cluster analysis generated two groups of Brazilian municipalities. DoD rates are consistently higher in the group that experiences more deprivation. However, considering differences between 2010 and 2019, the increase in rates was higher in the group with less deprivation experience (48.82 % vs. 39.53 %) We verified an existing gap between the clusters before the beginning of economic stagnation in 2010 The gap between those two groups decreased from 20.58 % (p < 0.001) in 2010 to 14.03 % in 2019 (p = 0.034). The conjuncture of economic crises creates mortality differentials in certain population groups. Also, significant inequalities explain how causes of death from despair affect different subpopulations. Our first approach assessed this assumption, and we could check those differentials at an ecological level. Public policies should focus on reducing the difference in mortality from despair between higher and lower socioeconomic strata.

## 1 Introduction

Population health results from the complex interaction between political, social, and economic dimensions. Most likely, social and economic conditions shape the lives people can lead (Marmot, 2017). Thus, an adverse combination of weak social programs and policies, unfair economic arrangements, and restrictive policies give rise to unequal health experiences (Marmot, 2022).

Over the past few years, there has been a progressive increase in deaths attributable to suicide, drug overdose, and alcohol-related liver disease in the US over the last two decades (Beseran et al., 2022). The pioneering study in this regard, carried out by Anne Case and Angus Deaton, coined the term “deaths from despair” (DoD) to characterize a theoretical framework that associates this trend with the economic crisis and the austerity measures taken to protect nations (Case and Deaton, 2015). The differentials pointed out by the original study, which induced several studies in the USA, indicate that there are relevant social determinants and inequalities related to these mortality trends.

Knapp et al. (2019), when analyzing deaths from despair in American counties, found that counties that experienced high economic insecurity in 2000 and 2010 had higher rates of deaths from despair and all-cause mortality in middle age in line baseline, with a difference of up to 41 % (95 % CI 36–47 %) between tertiles of economic insecurity. However, they showed similar rates of increase in deaths from despair from 2001 to 2015 compared to counties with stable low economic insecurity. Exploring long-term patterns in cause-specific mortality and mortality inequalities in Scotland, Allik et al. (2020) showed an increase in external cause mortality since the mid-1980 s accelerating in the early 1990 s and reaching a peak in the 2000 s. The pattern is drug deaths and suicides. Furthermore, they found that there has been a recent increase in absolute inequalities in mortality rates among young men according to the quintiles of economic deprivation classification, driven by external causes of mortality. Converging with this finding, Walsh et al. found a cohort effect in the Scottish data and the period and age effect highlighted by Allik et al. (2020). Hence, in Scotland, vulnerable cohorts of socio-economically deprived adults proved to be at greater risk, with essential responses to the social and political changes of the 1980 s.

Literature on DoD is still diverse regarding causal relationships with proximal determinants such as race and education (Augarde et al., 2022). Concerning race, the original study by Case and Deaton points to excess deaths among white men. In other contexts, migrants are penalized, and blacks are disadvantaged for deaths from despair (Beseran et al., 2022, Blair and Siddiqi, 2022)). As for education, the unavailability of data on educational status in death records, especially in countries with more significant heterogeneity and higher occurrence of low education, makes the evidence still fragile.

Nevertheless, there is a relative consensus on the relationship between sex and age groups, notably among middle-aged men (Tilstra et al., 2021). Regarding the relationship with contextual effects, the evidence is developing (Probst & Rehm, 2018), especially in countries with a peripheral economy, as is the case of Brazil. It is worth mentioning that Brazil has almost 6 thousand municipalities and significant heterogeneity among them (Lavinas and Simões, 2017). That said, we carried out an analysis whose objective was to estimate the relationship between socioeconomic insecurity and deaths due to despair in Brazilian cities.

## 2 Methods

We designed an ecological study to explore dimensions that the literature associates with despair. We used the following socioeconomic indicators: unemployment rate of people aged 18 or over; degree of formalization of those employed aged 18 or over; income ratio (ratio between the income of the wealthiest 10 % and the poorest 40 %); proportion of extremely poor people (whose average income is less than ¼ of the minimum wage (In 2010, the reference value of the minimum wage was US$ 294); percentage of population living in urban area; and Human Development Index.

We extracted data from socioeconomic indicators for the year 2010. We assume that the profile of municipalities for that year was the counterfactual scenario immediately prior to the onset of Brazilian economic stagnation caused by the global economic crisis of 2008. Fortunately, these are data from the last Brazilian Demographic Census, which offers an accurate picture of the situation in each municipality.

### 2.1 Statistical analysis

We performed a cluster analysis. Our objective was to classify Brazilian municipalities according to socioeconomic dimensions. We used the Euclidean distance as a measure of dissimilarity for grouping municipalities. The distance given by the Euclidean distances corresponds to the distance between entities Xi, and Xj.

$$d_{ij}=\left[X_{i}-X_{j}\right]={\left[\sum_{k=1}^{p}{\left(X_{i,k}-X_{j,k}\right)}^{2}\right]}^{1/2}$$

where p and k are given a matrix X in p-dimensional space.

We perform k-means clustering. From the previously specified number of 2 clusters, we defined the composition of the clusters with minimum intra-cluster total variation and maximum inter-cluster variation, considering the centroids of each group. For this, we use the Hartigan-Wong algorithm, which defines the total variation within the cluster as the sum of the squared distances of the Euclidean distances between the items and the corresponding centroid:

$$C\left(C_{k}\right)=\;\sum_{x_{eu}\in \;C_{k}}{\left(x_{eu}-\mu_{k}\right)}^{2}$$

where:

$x_{eu}$ is a data point belonging to the cluster.$C_{k}$

$\mu_{k}$ is the average value of the points assigned to the cluster.$C_{k}$

We compared the rates of death from despair of the formed clusters. We used the ICD-10 codes to estimate the number of deaths due to despair corresponding to the following groups of causes of death: accidental or intentional poisoning and poisoning of undetermined intent from drug exposure; drugs in the blood; drug-induced illnesses; mental/behavioral disorders due to drugs; alcohol-induced conditions; mental/behavioral disorders due to alcohol; accidental or intentional poisoning and poisoning of undetermined intent from alcohol exposure; suicide (complete list of codes available in Supplementary Material #1).

Our study hypothesis is that there is a difference in the rates so that the clusters with the worst performance of the indicators have a higher mortality rate. Formally, we define the hypothesis:

H_1_ = ASMR_i_ > ASMR_j_;
H_0_ = ASMR_i_ > ASMR_j_.

where ASMR is the death rate from despair in the i…j groups for 2019, adjusted by age group. We performed the standardization by the direct method, and we considered the Brazilian population of 2010 as the standard population. We used the ANOVA test to compare rates and obtain descriptive test statistics. We assumed a significance of 5 %. We performed the analysis using SPSS version 24.

We use secondary data of public and universal access, whose sensitive information is confidential and not available. In compliance with the Declaration of Helsinki, the study is exempt from evaluation by ethics committees. We address we have an institutional letter exempting it from peer evaluation.

## 3 Results

The 2010 socioeconomic indicators database had data for 5484 municipalities. The mortality database for 2019 had 5570 municipalities. This difference results from creation of 86 municipalities over the last ten years in Brazil. These municipalities were minor and excluded from the analysis, so we had data on the independent and dependent variables for all observations.

Cluster analysis generated two groups of Brazilian municipalities. The first cluster is composed of 3089 municipalities and the second cluster includes 2395 municipalities. When we compare the characteristics of the two groups, we can see that their profile differs entirely from each other. The first cluster showed a higher HDI (0.71 vs. 0.59), and a lower percentage of people living in rural areas more urbanized than cluster 2 (25.83 % vs. 49.36 %) (Table 1). Concerning working conditions, the municipalities in this cluster have a lower rate of adult unemployment and a higher degree of formalization of the working population. Regarding income and inequality indicators, for cluster 1 there is a less income inequality, and a smaller population living in extreme poverty. The difference between clusters is statistically significant for all indicators.

**Table 1:** Sociodemographic characteristics and magnitude of deaths of despair according to grouping of municipalities. Brazil, 2019.

**Variables**	**Cluster #1**		**Cluster #2**		**Total**		**p-value**
	**Mean**	**SD**	**Mean**	**SD**	**Mean**	**SD**	
Unemployment Rate over 18 years	5.60	0.60	7.08	0.73	6.25	0.48	<0.001
Formal Employees over 18 years (%)	57.59	2.06	24.83	1.54	43.29	1.71	<0.001
Income Ratio (10 % wealthiest | 40 % poorest)	11.46	0.42	18.45	0.71	14.51	0.51	<0.001
% of extreme poverty	3.47	0.60	21.84	2.18	11.47	1.63	<0.001
% of population living in urban area	74.17	8.40	50.64	6.0.61	63.69	7.01	<0.001
HDI	0.71	0.01	0.59	0.02	0.66	0.01	<0.001
	**ASMR**	**95 % CI**	**ASMR**	**95 % CI**	**ASMR**	**95 % CI**	**p-value**
Deaths of Despair (2010)	6.80	6.23–7.35	8.20	7.47–8.93	8.03	7.52–8.54	<0.001
Deaths of Despair (2019)	10.12	9.23–11.02	11.54	10.29–12.79	11.23	10.50–11.97	0.034

Notes: ASMR = Age-Standardized Mortality Rate; 95 % CI = 95 % Confidence Interval; SD = Standard Deviation; HDI = Human Development Index.

The rate of death from despair has a significant difference between the clusters. We calculated the age-adjusted mortality rate for 2010 and 2019. Those years correspond to a time before economic crisis and after crisis, recession, and fiscal austerity measures. We verified an existing gap between the clusters before the beginning of economic stagnation in 2010 (20.58 %, p < 0.001). This difference decreased to 14.03 % in 2019 (p = 0.034). To sum up, DoD rates are consistently higher in the group that experiences more deprivation. However, considering differences between 2010 and 2019, the increase in rates was higher in the group with less deprivation experience (48.82 % vs. 39.53 %).

## 4 Discussion

The conjuncture of economic crises creates mortality differentials in certain population groups. In turn, these differentials tend to be selective for specific causes of death (Glonti et al., 2015). Furthermore, significant inequalities explain how causes of death from despair affect different subpopulations. Our first approach assessed this assumption, and we could check those differentials at an ecological level. A study on the impact of the 2008 global economic crisis on suicide trends reveals an increase in mortality rates in European and American countries after the 2008 economic crisis. Increases in suicide occurred mainly among men and in countries with higher levels of job loss (Chang et al., 2013). We have a phenomenon that needs attention and further study since this behavior is probably related to these communities' underlying social and economic factors (Scutchfield and Keck, 2017).

Attempts to reduce external causes of mortality have focused on isolated causes of death. They have not effectively reduced mortality or inequalities from external causes in the long term (Allik et al., 2020). In general, context effects such as economic conditions, lower levels of education, working in highly precarious jobs, being unemployed, and living in rural areas appear as the most relevant social determinants of health with an impact on the rates of deaths due to despair (Beseran et al., 2022). Using a conceptual framework involving clinical and social aspects can contribute to the systemic approach to this public health problem. These robust theoretical models make it possible to explain the risk mechanisms for adverse mental health outcomes and to identify targets for clinical and collective intervention (Rehder et al., 2021). It includes addressing the social determinants of health and adopting equitable public policies.

Recent research has implicated economic insecurity in rising midlife mortality rates and “deaths of despair,” including suicide, chronic liver disease, and drug and alcohol poisoning. With an ecological design, the study by Knapp et al. (2019) highlights that economic insecurity may represent a factor driving mortality trends in the US at the population level. In the same direction, Probst et al. (2021) identified from a systematic review a dose–response relationship between low socioeconomic status (SES) and risk of mortality attributable to alcohol. Their analyses focused on socioeconomic deprivation indicators independent of educational level, income, and occupation. Regarding the difference in rates, we believe there was an increase in deaths from despair regardless of context. As we are comparing two periods with a difference of 10 years between them, we hypothesize that the growth rate was disproportionate between the clusters. This result converges with the findings of Knapp et al. (2019). It should be noted, however, that suicides account for 85 % of deaths due to despair in Brazil. Furthermore, it is essential to recognize that chronic liver disease can have a latency time much longer than the period covered by the study. That said, we believe that the difference in underlying causes did not bias the analysis.

Still, the sociodemographic homogeneity at various levels between individuals and communities, which Pescosolido et al. (2020) call “social similarity” or “similarity”, is little explored in the literature. Our study seeks to explore context variables, but the gain in using multilevel models for more consistent explanations is undeniable. The adoption of more complex models will allow the interaction between these levels of effects, and we will be able to assess whether they are concurrent risks or have synergistic effects. Possibly, there is a joint influence of both.

The fragility of the social protection system and the country's structural inequality plays an important role in explaining this type of association in Brazil. Borrell and cols analysys (Borrell et al., 2017, Borrell et al., 2021) suggest that, although the common association is “counties with the best performance regarding social indicators had lower ASMR related to despair”, when there is a financial or economic crisis, this pattern changes and it is not visible. The increase of death of despair is a short-term consequence of the economic crisis, with a clear increase in areas with the best performance regarding social indicators. We agree with this assumption. In fact, this analysis is correct and is in line with the recent explanation of this phenomenon in the USA. Many academic credit the low resilience of a subpopulation surrounded by privilege, such as middle-aged white men, with increased suicides and intoxications. This is even a point of criticism of Case and Deaton's work, which explores the more recent history, contrary to the silence that occurred years ago with the crack epidemic, which had a much stronger impact on the black population.

After improved our cluster model, we found results on the same direction. The group that experiences more deprivation kept higher mortality rates, However, considering differences between 2010 and 2019, the increase in rates was higher in the group with less deprivation experience. Nonetheless, we, we emphasize that the Brazilian context differs from that experienced by the studies by Borrel and collaborators. In Brazil, the characteristic of public policies, although intended to reduce disparities, first favor the middle class and increase the disparity between classes, penalizing the most vulnerable. Studies applied to health call this the Reverse Equity Theory (Victora et al., 2018). We believe that this theory applies to the present study, so we did verify the same association effect suggested by Borrell et al, but less impacted from that.

On the other hand, regarding the opioid epidemic, it is true that there is little relationship between the opioid crisis and contemporary measures of opportunities in the labor market. Cohorts and areas that have experienced poor labor market conditions show lagged increases in opioid mortality, but the effect is modest relative to the scale of the epidemic. Janet Currie and Hannes Schwandt (Currie & Schwandt, 2021) describe this effect, and we agree with their argument. However, we believe that in Brazil, this effect is not apparent since we have not yet experienced an epidemic of opioid use in the country. More recently, the Brazilian press has reported an increase in use, but it is recent and much later than the analysis period of our study.

Finally, we highlight that the most recent results in the literature suggest a potential impact of the Covid-19 pandemic on deaths due to despair, mainly due to suicide and overdose (Rahimi-Ardabili et al., 2022). This relationship occurs due to prolonged isolation and worsening economic crises that were already present in some countries before the pandemic. We must remember that this scenario affects minority groups, women, and vulnerable populations differently. For this reason, we chose to carry out the analysis in Brazil in the year immediately prior to the pandemic, when there was already a very adverse economic and political scenario in the country. Other studies after this preliminary analysis may bring greater robustness to the analyses, including more complex modeling that considers the temporal effect, the cohort effect, and the effect of the period marked by the course of the pandemic in the last three years.

The study has limitations. Our intention in discussing the clusters was to verify the effect of context. We used age-adjusted mortality rates to dilute the impact of the difference in age structure. It is true that eventually, there is a difference in composition by sex. However, our aim was not to verify individual factors. Recognizing this limitation, we suggest that the results be viewed with caution, and the ecological study, as indicated by its design, only raises diagnostic hypotheses to be tested in due course, preferably with techniques that concomitantly assess individual and contextual effects, such as hierarchical models. We also recognize that mortality trends in the three causes may not necessarily share the exact underlying causes, as cited by Masters et al. (2017).

In the end, it is likely that social and economic circumstances are the main contributors to the observed increase in specific groups and the significant differences in rates by race or social class. Therefore, public policies should focus on reducing the difference in mortality from despair between higher and lower socioeconomic strata. The next step is to determine what can be done to reverse the worrying rise in death rates from despair and eliminate the deep health inequalities by race, social class, and geography that have characterized the Brazilian social structure. For this, the following approaches should consider including individual and contextual variables using multilevel modeling.

## 6 Ethical aspects

We use secondary data of public and universal access, whose sensitive information is confidential and not available. In compliance with the Declaration of Helsinki, the study is exempt from evaluation by ethics committees.

## Funding

The authors did not receive support from any organization for the submitted work.

## CRediT authorship contribution statement

**Wellington Roberto Gomes de Carvalho:** Formal analysis, Investigation, Writing – original draft. **Karina Cardoso Meira:** Visualization, Writing – original draft, Writing – review & editing. **Aline Grimberg Pereira de Medeiros:** Visualization, Writing – original draft, Writing – review & editing. **Raphael Mendonça Guimarães:** Conceptualization, Data curation, Formal analysis, Methodology, Visualization, Writing – original draft, Writing – review & editing.

## Declaration of competing interest

The authors declare that they have no known competing financial interests or personal relationships that could have appeared to influence the work reported in this paper.

## Data Availability

This study analyzed secondary public data. All indicators are available at: http://www.atlasbrasil.org.br/. All mortality data are available at https://datasus.saude.gov.br/home/tabnet/
