# Supplementary material for: Socio-economic vulnerability and deaths of despair in Brazilian counties
Source: Prev Med Rep. 2024 Jan 20;38:102623. doi: 10.1016/j.pmedr.2024.102623 (PMC10874878; doi:10.1016/j.pmedr.2024.102623)
Supplement: Supplementary Data 1 [file mmc1.docx]

**Supplementary File #1**

**Table:** Causes of Death and ICD-10 codes related to deaths of despair.

| **Group of causes** | **ICD-10 codes** |
| --- | --- |
| Accidental or intentional poisoning and poisoning of undetermined intent from drug exposure; drugs in the blood | X40-X44, X60-64, Y10-Y14; R78.1-R78.5 |
| Drug-induced illnesses | D52.1, D59.0, D59.2, D61.1, D64.2, E06.4, E16.0, E23.1, E24.2, E27.3, E66.1, G21 .1, G24.0, G25.1, G25.4, G25.6, G44.4, G62.0, G72.0, I95.2, J70.2-J70.4, K85.3, L10.5 , L27.0, L27.1, M10.2, M32.0, M80.4, M81.4, M83.5, M87.1, R50.2 |
| Mental/behavioral disorders due to drugs | (F11.0-F11.5, F11.7-F11.9, F12.0-F12.5, F12.7-F12.9, F13.0-F13.5, F13 .7-F13.9, F14.0-F14.5, F14.7-F14.9, F15.0-F15.5, F15.7-F15.9, F16.0-F16.5, F16.7 -F16.9, F18.0-F18.5, F18.7- F18.9, F19.0-F19.5, F19.7-F19.9 |
| Alcohol-induced illnesses | E24.4, G31.2, G62.1, G72.1, I42.6, K29.2, K70.0-K70.4, K70.9, K73.4, K85.2, K86.0, R78.0 |
| Mental/behavioral disorders due to alcohol | F10.0-F10.9 |
| Accidental or intentional poisoning and poisoning of undetermined intent from alcohol exposure | X40-45, X65, Y10-15, Y45, Y47, Y49 |
| Suicide | X66-X84, Y87.0 |
